# Stereoselectivity Switch in the Reduction of α-Alkyl-β-Arylenones by Structure-Guided Designed Variants of the Ene Reductase OYE1

**DOI:** 10.3389/fbioe.2019.00089

**Published:** 2019-04-26

**Authors:** Michele Crotti, Fabio Parmeggiani, Erica Elisa Ferrandi, Francesco G. Gatti, Alessandro Sacchetti, Sergio Riva, Elisabetta Brenna, Daniela Monti

**Affiliations:** ^1^Dipartimento di Chimica, Materiali e Ingegneria Chimica “G. Natta”, Politecnico di Milano, Milan, Italy; ^2^Istituto di Chimica del Riconoscimento Molecolare, C.N.R., Milan, Italy

**Keywords:** ene reductases, old yellow enzymes, protein engineering, biocatalysis, stereoselectivity, reduction

## Abstract

Ene reductases from the Old Yellow Enzyme (OYE) family are industrially interesting enzymes for the biocatalytic asymmetric reduction of alkenes. To access both enantiomers of the target reduced products, stereocomplementary pairs of OYE enzymes are necessary, but their natural occurrence is quite limited. A library of wild type ene reductases from different sources was screened in the stereoselective reduction of a set of representative α-alkyl-β-arylenones to investigate the naturally available biodiversity. As far as the bioreduction of the ethyl ketone derivatives concerns, the results confirmed the distinctiveness of the OYE3 enzyme in affording the reduced product in the (*S*) configuration, while all the other tested ene reductases from the Old Yellow Enzymes family showed the same stereoselectivity toward the formation of corresponding (*R*) enantiomer. A possible determinant role of the “hot spot” residue in position 296 for the stereoselectivity control of these reactions was confirmed by the replacement of Phe296 of OYE1 with Ser as found in OYE3. Further investigations showed that the same stereoselectivity switch in OYE1 could be achieved also by the replacement of Trp116 with Ala and Val, these experimental results being rationalized by structural and docking studies. Moreover, an additive effect on the stereoselectivity of OYE1 was observed when coupling the selected mutations in position 296 and 116, thus providing two extremely enantioselective variants of OYE1 (W116A-F296S, W116V-F296S) showing the opposite stereoselectivity of the wild type enzyme. Lastly, the effects of the mutations on the bioreduction of carvone enantiomers were investigated as well.

## Introduction

The application of enzymes, either isolated or as whole cells, as biocatalysts in organic synthesis is gaining increasing interest for the sustainable and efficient production of high-value products such as pharmaceutical intermediates and fine chemicals (Bornscheuer et al., 2012; Bornscheuer, 2018; Devine et al., 2018). In particular, the development of biocatalyzed processes looks markedly attractive when the alternative chemosynthetic routes are challenging and/or when a high chemo-, regio- or stereoselectivity is required.

Ene reductases (ERs) from the Old Yellow Enzyme (OYE) family (EC 1.6.99.1) are flavin mononucleotide (FMN)-containing oxidoreductases capable to catalyze the stereoselective reduction of α,β-unsaturated compounds activated by the presence of an electron-withdrawing group (EWG) in proximity of the C-C double bond, typically a carbonyl group or a nitro group (Gatti et al., 2013; Toogood and Scrutton, 2014, 2018; Knaus et al., 2016; Winkler et al., 2018). The catalytic activity of these enzymes results therefore in an asymmetric *anti*-hydrogenation reaction where up to two stereogenic centers could possibly be created in a single step. The catalytic mechanism of OYEs is well-understood: the enzyme-bound flavin is first reduced at the expense of NAD(P)H cofactor (reductive half-reaction), then a hydride is transferred from the reduced FMNH_2_ to the electronically activated Cβ position of the alkene substrate (Karplus et al., 1995; Toogood et al., 2010). The *in situ* regeneration of the reduced nicotinamide cofactor can be easily achieved in practical *in vitro* applications by using an enzymatic recycling system, e.g., that using a NAD(P)H-dependent glucose dehydrogenase (GDH) and glucose as a co-substrate.

The occurrence of ER activities is widespread in Nature and, thanks also to the ever growing available data from genomes sequencing, OYE homologs have been identified from various sources, such as bacteria, yeasts, fungi, and plants (Scholtissek et al., 2017). Many of these enzymes have been cloned during the last years, thus making them available in recombinant form for preparative-scale application in organic synthesis.

The synthetic utility of ERs has already been demonstrated in several cases, for example in the preparation of different chiral building blocks (Brenna et al., 2013c, 2015a, 2017b; Oberleitner et al., 2013; Monti et al., 2015; Turrini et al., 2017; Hadi et al., 2018), pharmaceutical intermediates (Korpak and Pietruszka, 2011; Pietruszka and Schölzel, 2012; Brenna et al., 2013b; Winkler et al., 2013; Debarge et al., 2014; Kelly et al., 2016), flavors (Bougioukou et al., 2010; Brenna et al., 2017a), and fragrances (Stueckler et al., 2010; Brenna et al., 2015b). Interestingly, in many of these examples the application of ERs has been successfully integrated in more complex multistep chemoenzymatic or multienzymatic cascade processes with significant advantages in terms of yields, selectivity and environmental impact (Toogood and Scrutton, 2018).

During the last years, the extensive investigations by different research groups on the biocatalytic application of ERs in the bioreduction of a large array of α,β-unsaturated substrates (e.g., enals, enones, nitroalkenes, acrylate esters, and acrylonitriles) highlighted their remarkable stereoselectivity. This outcome has been elucidated by the understanding of the binding modes of the substrates in the active site of wild type and engineered enzymes (Gatti et al., 2013; Amato and Stewart, 2015), that, alternatively, lead to the preferential (or exclusive) formation of either one or the other enantiomer of the reduced product, a phenomenon also referred to as facial selectivity (Walton et al., 2014). Additionally, substrate-based stereocontrol strategies, e.g., those using (*E*)/(*Z*)-stereo-/regioisomers or properly protected substrates, have been successfully applied in different cases to improve or switch the natural ERs stereoselectivity (Brenna et al., 2012; Oberdorfer et al., 2012).

However, the access to both enantiomers of a defined product by ERs-catalyzed bioreduction is still challenging, as most of the wild type OYEs show the same stereopreference. In fact, only a limited number of stereocomplementary pairs have been identified so far by screening of wild type ERs (Hall et al., 2007; Stueckler et al., 2011; Walton et al., 2011; Tasnádi et al., 2012; Brenna et al., 2017c) and protein engineering (Bougioukou et al., 2009; Padhi et al., 2009; Pompeu et al., 2013; Walton et al., 2014; Rüthlein et al., 2015; Kress et al., 2017; Nett et al., 2017).

**Figure 1:**
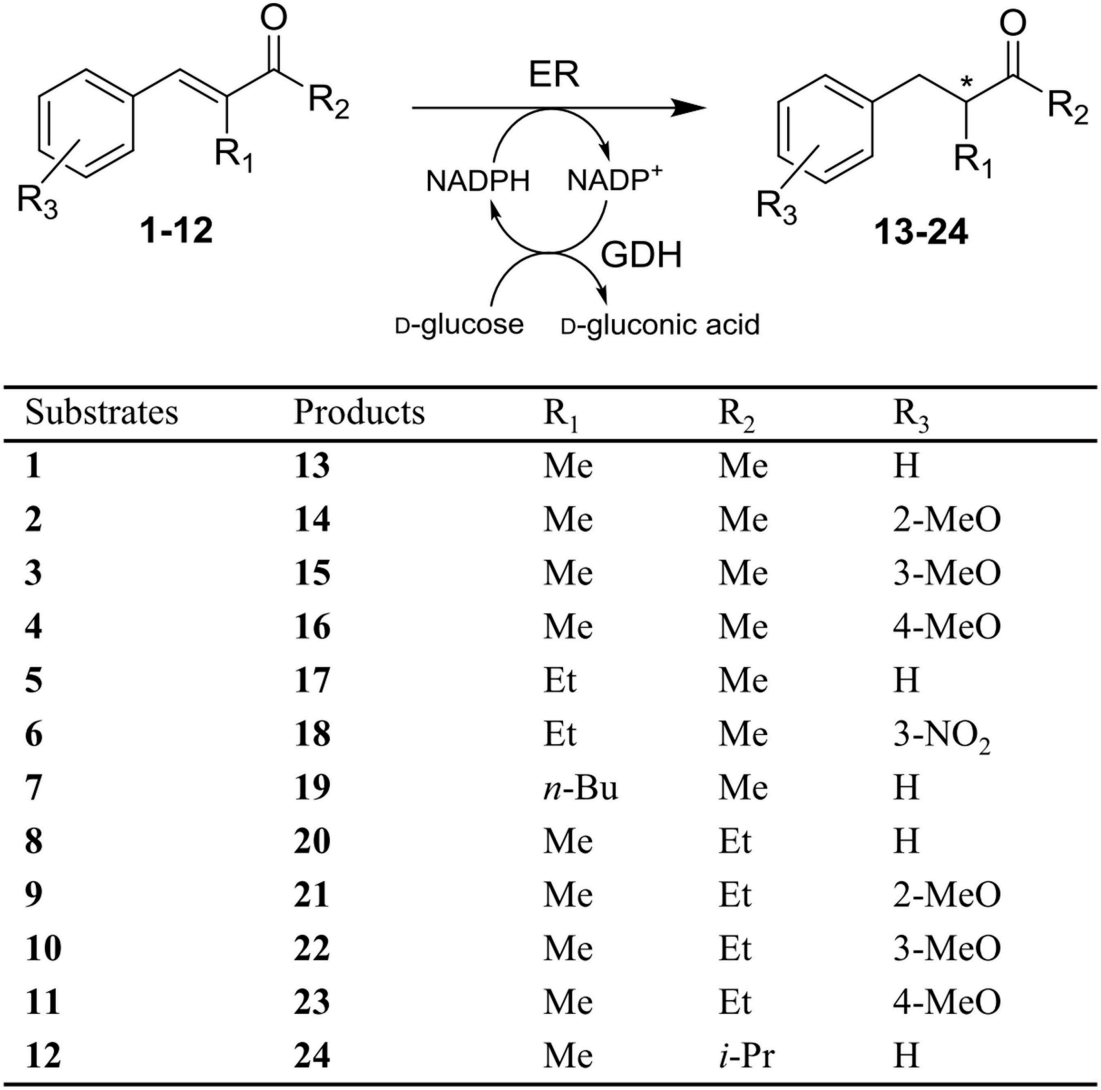
Stereoselective reduction of substrates **1-12** by ene reductases (ERs).

**Figure 2:**
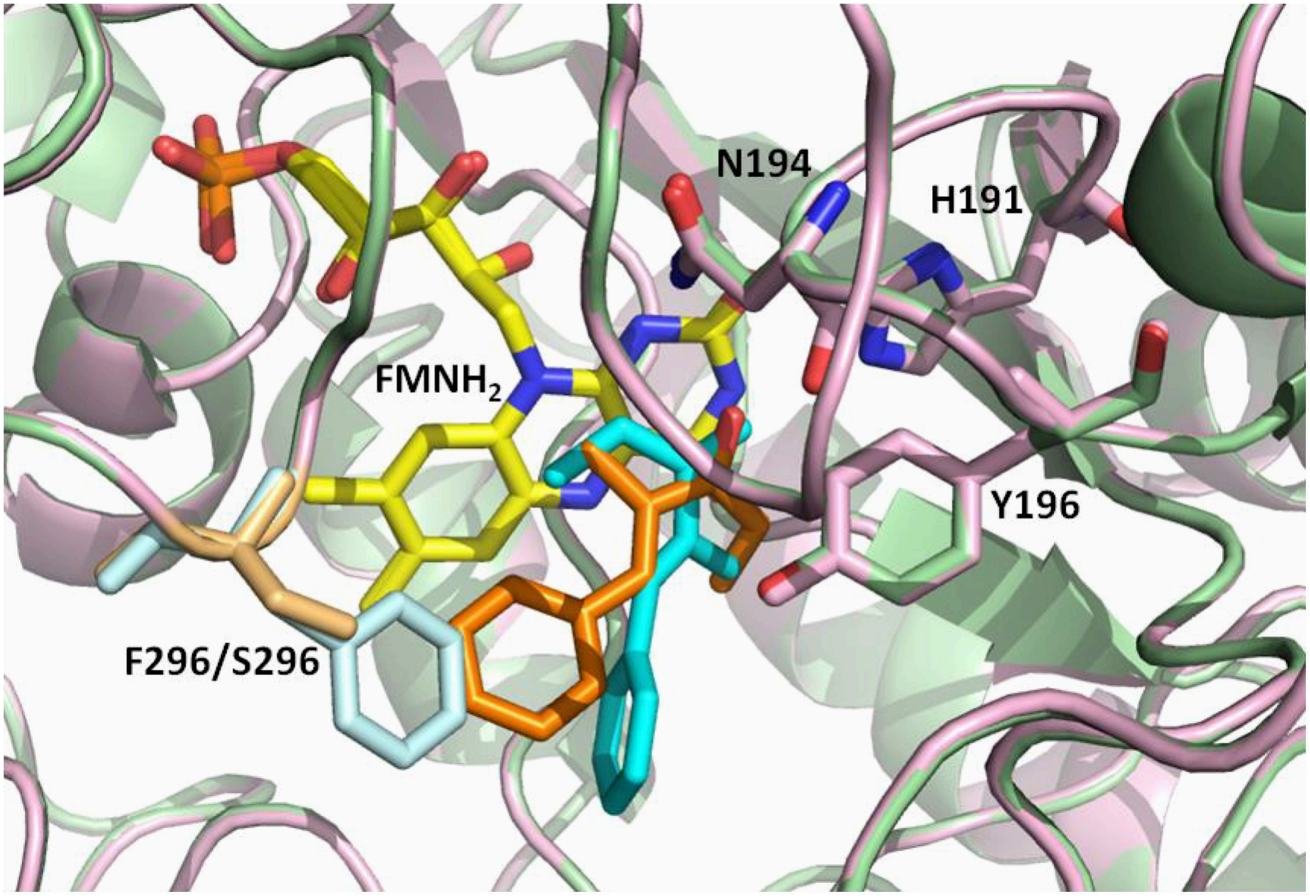
Overlay of the OYE1 and OYE3 active sites showing possible binding orientations of the ethyl ketone **8** (cyan in OYE1, orange in OYE3), as suggested by previous docking studies (Brenna et al., 2013a). Phe296 in OYE1 is shown in light blue, Ser196 in OYE3 in sand, while the FMNH_2_ cofactor is shown in yellow.

An interesting case of stereocomplementarity has been reported by our group some years ago when studying the OYE-catalyzed reduction of α-alkyl-β-arylenones (Brenna et al., 2013a). Specifically, in the case of the ethyl ketone **8** (Figure 1) and of its methoxy derivatives **10** and **11**, the enzymes OYE1 from *Saccharomyces pastorianus* and OYE2 from *S. cerevisiae* showed a preference in the formation of the (*R*)-enantiomer of the reduced products, while the isoform OYE3 from *S. cerevisiae* furnished the saturated products with (*S*) configuration in up to 95% enantiomeric excess (ee). The very high sequence similarity between these enzymes (80 and 82 % sequence identity at the amino acidic level between OYE3 and OYE1 or OYE2, respectively) suggested that this stereocomplementary behavior may be ascribed to different binding modes of the substrates due to subtle differences in the respective active sites (Figure 2) (Brenna et al., 2013a).

**Figure 3:**
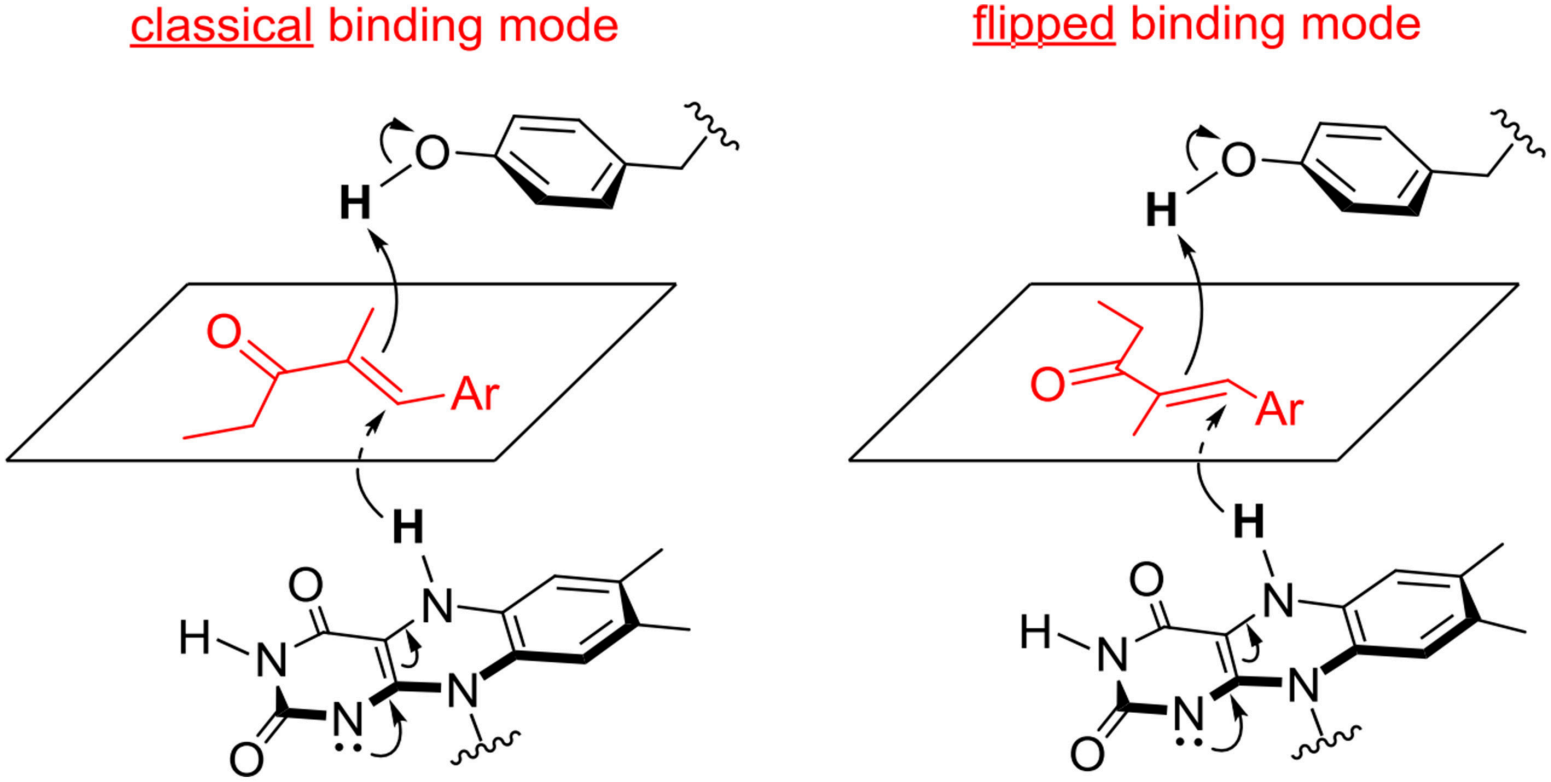
Possible binding modes of α,β-unsaturated ethyl ketones in the active site of OYE 1 (classical binding mode) and OYE 3 (flipped binding mode).

This hypothesis was recently investigated by Stewart et al. Labeling experiments were employed to confirm the *anti* stereochemistry of H_2_ addition to the C = C double bond of α,β-unsaturated ethyl ketones, and establish that the opposite enantioselectivity of their reduction with OYE1 and OYE3 was due to the exposure of one or the other face of the alkene moiety to hydride attack, as a consequence of different substrate binding modes (Figure 3). In the same work, the crystal structures of ligand-free and phenol-bound OYE3 were solved (PDB codes: 5V4V, 5V4P) (Powell et al., 2018). The comparison of OYE1 and OYE3 crystal structures confirmed their very high similarity, the only difference being the position of the side chain of the residue in position 296, pointing either toward the active site (Phe296 in OYE1) or toward the solvent (Ser296 in OYE3). Unfortunately, the obtainment of OYE3 crystal structure did not help to completely understand the role of Ser296 in the observed stereoselectivity, possibly due to the fact that this amino acid lies on a protein loop (loop 6) showing a different conformational mobility in OYE3 when compared to OYE1 (Powell et al., 2018).

Therefore, this work was aimed at further investigating the OYE-catalyzed bioreduction of α-alkyl-β-arylenones using first of all a larger set of wild type enzymes. The results obtained in this screening has led us to the design of selected OYE1 variants, whose synthetic potential and stereoselectivity have been experimentally investigated on different target substrates and rationalized by structural and docking studies.

## Materials and Methods

### General Methods

Chemicals and solvents were purchased from suppliers and used without further purification. ^1^H and ^13^C NMR spectra were recorded with a 400 MHz spectrometer at rt in CDCl_3_ solution (unless otherwise specified), using TMS as an internal standard for ^1^H and CDCl_3_ for ^13^C; chemical shifts δ are expressed in ppm relative to TMS. GC-MS analyses were performed on an Agilent HP 6890 gaschromatograph equipped with a HP-5MS column (30 m × 0.25mm × 0.25 μm), with *t*_inj_ = 250°C, injecting 1 μL of solution, and using He as a carrier gas (1.0 mL·min^−1^). Method: 60°C (1 min)/6°C/min/150°C (1 min)/12°C/min/280°C (5 min). Determination of enantiomeric excess of products **13**–**24** was performed by chiral GC analyses on a DANI HT 86.10 gas-chromatograph equipped with a Varian Chirasil-DEX-CB (25 m × 0.25 mm) column and a flame ionization detector, with *t*_inj_ = 250°C and *t*_det_ = 250°C, injecting 1 μL of solution and using H_2_ as a carrier gas (1.5 mL·min^−1^). (see Supporting Information for details).

### Bacterial Strains

Ene reductases OYE1-3 and glucose dehydrogenase (GDH) were produced in recombinant form in *Escherichia coli* BL21(DE3) strains harboring a specific plasmid prepared as previously reported: pET30a-OYE1 from the original plasmid provided by Prof. Neil C. Bruce (Williams et al., 2004), pET30a-OYE2 and pET30a-OYE3 from *S. cerevisiae* BY4741 and pKTS-GDH from *Bacillus megaterium* DSM509 (Bechtold et al., 2012). All other wild type ERs and the library of W116X OYE1 variants were obtained from recombinant *E. coli* BL21(DE3) strains transformed with the original pDJBx and pDJB5 plasmids, respectively (Bougioukou et al., 2010; Walton et al., 2011), both provided by Prof. Jon D. Stewart.

### Enzyme Expression, Purification and Site-Directed Mutagenesis

LB medium (100 mL) containing the appropriate antibiotic (50 μg·mL^−1^ kanamycin for pET30a, 100 μg·mL^−1^ ampicillin for pKTS, pDJBx, and pDJB5) was inoculated with freshly plated cells and grown at 37°C and 220 rpm overnight. The culture was then used to inoculate 750 mL of fresh medium and growth of recombinant *E. coli* was carried out at 37°C and 220 rpm until OD_600_ reached 0.4–0.5, then enzyme expression was induced by the addition of 0.1 mM IPTG (50 ng·mL^−1^ anhydrotetracycline was also added in the case of the pKTS-GDH plasmid). After about 4 h at 30°C (37°C in the case of pKTS-GDH), the cells were harvested by centrifugation (3000 × g, 20 min, 4°C).

In the case of OYE1-3 and GDH—produced as His-tagged proteins—the cell pellet was resuspended in the minimal volume (10–20 mL) of washing buffer (20 mM potassium phosphate (KP) buffer pH 7.0, 500 mM NaCl, 20 mM imidazole) and disrupted by sonication (Omni Ruptor 250 ultrasonic homogenizer, Omni International Inc., Kennesaw, GE, USA, five sonication cycles, 15 s each, 50% duty). The clear lysate obtained after centrifugation (3,000 × g, 40 min, 4°C) was incubated with 8 mL of the Ni Sepharose 6 Fast Flow agarose resin (GE Healthcare, Milano, Italy) at 4°C for 1–2 h under mild shaking. The resin was then loaded into a glass column (10 × 110 mm) and washed with 10 mL of washing buffer. Bound proteins were eluted using a 3 step gradient, washing the column with a mixture of washing buffer and elution buffer (20 mM KP buffer, pH 7.0, 500 mM NaCl, 500 mM imidazole) in 4:1, 3:2, and 2:3 ratio (8 mL each step), respectively. Fractions (2 mL) were collected, protein concentration was measured using the Bio-Rad Protein Assay and bovine serum albumin as a standard according to the Bradford method and the protein purity was verified by SDS-PAGE analysis (10% T, 2.6% C). When necessary, purified protein aliquots were dialysed twice against 1.0 L of 50 mM KP buffer pH 7.0 (12 h each, 4°C) to remove imidazole and salts, then stored at −80°C.

In the case of the other ER enzymes—produced as Glutathione S-Transferase (GST)-fusion proteins—cell pellets were resuspended in cold PBS binding buffer (140 mM NaCl, 2.7 mM KCl, 10 mM Na_2_HPO_4_, 1.8 mM KH_2_PO_4_, pH 7.3) and lysed by sonication (Omni Ruptor 250 ultrasonic homogenizer, five sonication cycles, 15 s each, 50% duty). The cell extract was recovered by centrifugation (3,000 × g, 40 min, 4°C) and incubated with 8 mL of Glutathione Sepharose 4 Fast Flow resin (GE Healthcare) at 4°C for 1–2 h under mild shaking. The resin was then loaded into a glass column (10 × 110 mm) and washed with 10 mL of PBS buffer, then GST-tagged enzyme elution was achieved by adding a reduced glutathione (GSH) buffer solution (10 mM γ-l-glutamyl-l-cysteinylglycine, 50 mM Tris-HCl, pH 8.0). Purified protein aliquots were kept at −80°C until use.

Site-directed mutagenesis of OYE1 was carried out with the QuikChange II XL Site-Directed Mutagenesis Kit (Qiagen) according to the manufacturer's instructions. A list of the mutagenic primers is available in (Table S1).

### General Procedure for ERs-Mediated Bioreductions

The enone (5 μmol) dissolved in DMSO (10 μL) was added to a phosphate buffer solution (1.0 mL, 50 mM, pH 7.0) containing glucose (20 μmol), NADP^+^ (0.1 μmol), GDH (200 μg mL^−1^, 4 U mL^−1^) and the required ER (40 μg mL^−1^). The mixture was incubated for 24 h in an orbital shaker (160 rpm, 30°C). The solution was extracted with EtOAc (2 × 250 μL), centrifuging after each extraction (15000 *g*, 1.5 min), and the combined organic solutions were dried over anhydrous Na_2_SO_4_ for further analysis.

### Computational Methods

The OYE1 enzyme was modeled starting from its x-ray structure (PDB code: 1OYB) as reported in a previous paper (Brenna et al., 2013a). The models for the mutants were obtained from the literature (Pompeu et al., 2013). The x-ray structure of the complexes of the enzyme with (*R*)-carvone were selected (PDB code: 4K7V, 4GEW and 4K8H). All the structures were prepared for the docking with the software YASARA. The ketone substrate **8** has been modeled after a conformational search with the Monte Carlo approach and the MMFF94 force field within the Spartan'08 software (Wavefunction, Inc. Irvine, CA). The most stable conformations were then optimized at the DFT (B3LYP-6-31Gd) level. Docking was performed using AutoDock (Morris et al., 2009) using the default docking parameters supplied with AutoDock in the “examples” subdirectory, and point charges initially assigned according to the AMBER03 force field (Duan et al., 2003), and then damped to mimic the less polar Gasteiger charges used to optimize the AutoDock scoring function. The setup was done with the YASARA molecular modeling program (Krieger et al., 2004; Krieger and Vriend, 2014). For each ligands 25 Autodock LGA runs were executed. Results were sorted by binding energy (more positive energies indicate stronger binding, and negative energies mean no binding). After clustering the 25 runs, the resulting complex conformations were originated and clustered (they all differ by at least 5.0 Å heavy atom RMSD).

## Results

### Screening of a Library of Wild Type Ene Reductases in the Bioreduction of α,β-Unsaturated-β-Arylketones

In the first part of this work, the screening of the stereoselective reduction of α-alkyl-β-arylenones (Figure 1), previously carried out with the OYE1-3 enzymes (Brenna et al., 2013a), was extended to a broader set of ERs (Table 1). Specifically, seven different ERs belonging to the OYE family and from different sources (bacteria, plants, and yeasts, see also Table S2) and LtB4DH, a NADPH-dependent flavin-free alkene reductase from *Rattus norvegicus* belonging to the medium-chain dehydrogenase superfamily (Bougioukou and Stewart, 2008), were included in the screening to improve the biocatalysts diversity. In fact, the sequence identities between the novel OYEs and OYE1-3 range from 33 to 72% (see Table S3).

**Table 1:** Sources and sequences of ene reductases (ERs) used in this study.

**ER**	**Source**	**Species**	**UniProtKB accession number**
OYE1	Yeast	*Saccharomyces pastorianus*	Q02899
OYE2	Yeast	*Saccharomyces cerevisiae*	Q03558
OYE3	Yeast	*Saccharomyces cerevisiae*	P41816
NemA	Bacterium	*Escherichia coli*	P77258
OPR1	Plant	*Arabidopsis thaliana*	Q8LAH7
OYE2.6	Yeast	*Scheffersomyces stipitis*	A3LT82
PpNemA	Bacterium	*Pseudomonas putida*	Q88I29
LeOPR1	Plant	*Solanum lycopersicum*	Q9XG54
KmOYE	Yeast	*Kluyveromyces marxianus*	Q6I7B7
LtB4DH	Animal	*Rattus norvegicus*	P97584
PpOYE	Bacterium	*Pseudomonas putida*	Q88K07

The bioreductions were performed using purified ERs and the *in situ* regeneration of the NADPH cofactor was performed using glucose dehydrogenase (GDH) in the presence of glucose as a sacrificial cosubstrate. Samples of the reaction mixtures were extracted with organic solvent after 24 h and the conversion was determined by GC-MS analyses, while the enantiomeric excess (ee) values of the reduced products were obtained by GC analyses on a chiral stationary phase.

As shown in Table 2, all the selected substrates could be reduced by most or all of the ERs with the exclusion of substrates **9** and **12**, for which the conversion degree was negligible under all the tested conditions, thus confirming the detrimental effect on the bioreductions of the presence of bulky substituents at the R_2_ positions, especially when combined with the presence of a methoxy group in *ortho* position on the aromatic ring. The same effect was observed also with substrate **7** bearing a butyl chain at the α-position (R_1_). Very little or no conversion was observed with the plant enzyme OPR1 and the bacterial ER PpOYE, the latter showing almost quantitative conversion only with the *p*-methoxy methyl derivative **4**. The other ERs showed a good to excellent activity toward various substrates with some differences in terms of substrate preference. The non-OYE reductase LtB4DH demonstrated a very good activity toward most substrates, but the reduced products generally showed quite low ee values. Instead, a remarkable stereoselectivity was shown by the yeast ER OYE2.6, in particular in the case of the ethyl ketone **8** and of its derivatives **10** and **11**, where the (*R*)-enantiomer of the reduced products could be obtained with up to 99% ee values.

**Table 2:** Screening of ERs-catalyzed bioreduction of α,β-unsaturated-β-arylketones.

**ER^**a**^**	**Substrate**									
	**1**	**2**	**3**	**4**	**5**	**6**	**7**	**8**	**10**	**11**
	***c* [%]^**b**^**	***c* [%]^**b**^**	***c* [%]^**b**^**	***c* [%]^**b**^**	***c* [%]^**b**^**	***c* [%]^**b**^**	***c* [%]^**b**^**	***c* [%]^**b**^**	***c* [%]^**b**^**	***c* [%]^**b**^**
	***ee* [%]^**c**^**	***ee* [%]^**c**^**	***ee* [%]^**c**^**	***ee* [%]^**c**^**	***ee* [%]^**c**^**	***ee* [%]^**c**^**	***ee* [%]^**c**^**	***ee* [%]^**c**^**	***ee* [%]^**c**^**	***ee* [%]^**c**^**
OYE1^d^	>99	>99	>99	92	>99	>99	16	67	53	41
	56 (*S*)	97 (*S*)	95 (*S*)	42 (*S*)	99 (*S*)	99 (*S*)	98 (*S*)	59 (*R*)	20 (*R*)	60 (*R*)
OYE2 ^d^	>99	61	>99	64	52	73	3	9	11	25
	61 (*S*)	95 (*S*)	97 (*S*)	59 (*S*)	99 (*S*)	99 (*S*)	n.d.^e^	76 (*R*)	42 (*R*)	69 (*R*)
OYE3 ^d^	>99	46	>99	45	>99	88	6	14	8	7
	98 (*S*)	96 (*S*)	99 (*S*)	97 (*S*)	99 (*S*)	99 (*S*)	n.d.^e^	89 (*S*)	95 (*S*)	81 (*S*)
NemA	53	2	16	18	95	23	2	16	4	1
	58 (*S*)	62 (*S*)	78 (*S*)	77 (*S*)	90 (*S*)	84 (*S*)	n.d.^e^	66 (*R*)	36 (*R*)	40 (*R*)
OPR1	1	–	–	<1	–	–	–	–	2	–
	n.d.^e^	n.d.^e^	n.d.^e^	n.d.^e^	n.d.^e^	n.d.^e^	n.d.^e^	n.d.^e^	99 (*R*)	n.d.^e^
OYE2.6	>99	15	99	83	>99	84	0.5	>99	60	67
	82 (*S*)	46 (*S*)	86 (*S*)	54 (*S*)	99 (*S*)	99 (*S*)	n.d.^e^	99 (*R*)	88 (*R*)	99 (*R*)
PpNemA	7	–	30	7	20	71	–	–	2	–
	66 (*S*)	n.d.^e^	72 (*S*)	n.d.^e^	99 (*S*)	98 (*S*)	n.d.^e^	n.d.^e^	94 (*R*)	n.d.^e^
LeOPR1	2	2	40	7	87	30	5	5	2	–
	n.d.^e^	1 (*S*)	56 (*S*)	n.d.^e^	86 (*S*)	90 (*S*)	n.d.^e^	30 (*R*)	74 (*R*)	n.d.^e^
KmOYE	>99	11	99	95	86	72	0.7	8	11	5
	18 (*S*)	86 (*S*)	92 (*S*)	54 (*S*)	99 (*S*)	99 (*S*)	n.d.^e^	88 (*R*)	24 (*S*)	80 (*R*)
LtB4DH	>99	27	99	1	>99	84	6	68	94	95
	14 (*S*)	13 (*S*)	24 (*S*)	n.d.^e^	22 (*S*)	33 (*S*)	n.d.^e^	10 (*S*)	64 (*S*)	36 (*S*)
PpOYE	1	–	4	98	2	–	–	–	1	–
	n.d.^e^	n.d.^e^	60 (*S*)	18 (*S*)	n.d.^e^	n.d.^e^	n.d.^e^	n.d.^e^	94 (*R*)	n.d.^e^

a *See Table S2 for enzyme source and sequences*.

b *Conversion values determined by GC-MS*.

c *Enantiomeric excess values determined by GC on a chiral stationary phase. Reactions were performed in triplicate, standard deviation of conversion and enantiomeric excess values was below 5%*.

d *Reported from Brenna et al. (2013a)*.

e *n.d., Not determined, below detection limit*.

Interestingly, this screening confirmed that the stereopreference switch previously observed with OYE3 with the enones **8**, **10**, and **11** could not be achieved with any of the other ene reductases of the OYE family, which share with OYE1 and OYE2 the presence of a phenylalanine at position 296.

### Structure-Guided Design of OYE1 Variants

The results obtained during the screening prompted us to further investigate the role of the key active site residue F296 in OYE1 in the stereochemical control of the bioreductions.

Firstly, the replacement of F296 in OYE1 with a serine residue, as found in OYE3, was performed by site-directed mutagenesis. The activity and stereoselectivity of the generated OYE1 variant in the bioreduction of a selected set of α,β-unsaturated methyl and ethyl ketones was compared with those of the wild type ERs OYE1-3 (Table 3, lines 1-4).

**Table 3:** Bioreduction of selected α,β-unsaturated methyl and ethyl ketones by wild type and mutant ERs.

**Entry**	**ER**	**Substrate**							
		**1**		**8**		**10**		**11**	
		***c* [%]^**a**^**	***ee* [%]^**b**^**	***c* [%]^**a**^**	***ee* [%]^**b**^**	***c* [%]^**a**^**	***ee* [%]^**b**^**	***c* [%]^**a**^**	***ee* [%]^**b**^**
1	OYE1	>99	56 (*S*)	67	59 (*R*)	53	20 (*R*)	41	60 (*R*)
2	OYE2	>99	61 (*S*)	9	76 (*R*)	11	42 (*R*)	25	69 (*R*)
3	OYE3	>99	98 (*S*)	14	89 (*S*)	8	95 (*S*)	7	81 (*S*)
4	OYE1 F296S	>99	94 (*S*)	6	44 (*S*)	10	80 (*S*)	5	34 (*S*)
5	OYE1 F296S/W116A	35	90 (*S*)	18	96 (*S*)	27	98 (*S*)	3	92 (*S*)
6	OYE1 F296S/W116V	57	94 (*S*)	19	94 (*S*)	19	98 (*S*)	3	90 (*S*)

a *Conversion values determined by GC-MS*.

b *Enantiomeric excess values determined by GC on a chiral stationary phase. Reactions were performed in triplicate, standard deviation of conversion and enantiomeric excess values was below 5%*.

The OYE1 F296S mutant conserved a very good activity toward the methyl derivative **1** showing an even improved *S*-selectivity, with ee values for the reduced product going from 56% with the wild type enzyme to 94% with the variant. Instead, the results obtained with the ethyl ketones **8**, **10**, and **11** supported our starting hypothesis, since an enantioselectivity switch was observed, leading to the preferential formation of the *S* enantiomer instead of the *R* product as observed with the wild type OYE1. However, the ee values were generally lower than those obtained with OYE3, the best selectivity being observed in both cases with substrate **10** (80 and 95% ee with OYE1 F296S and OYE3, respectively).

**Figure 4:**
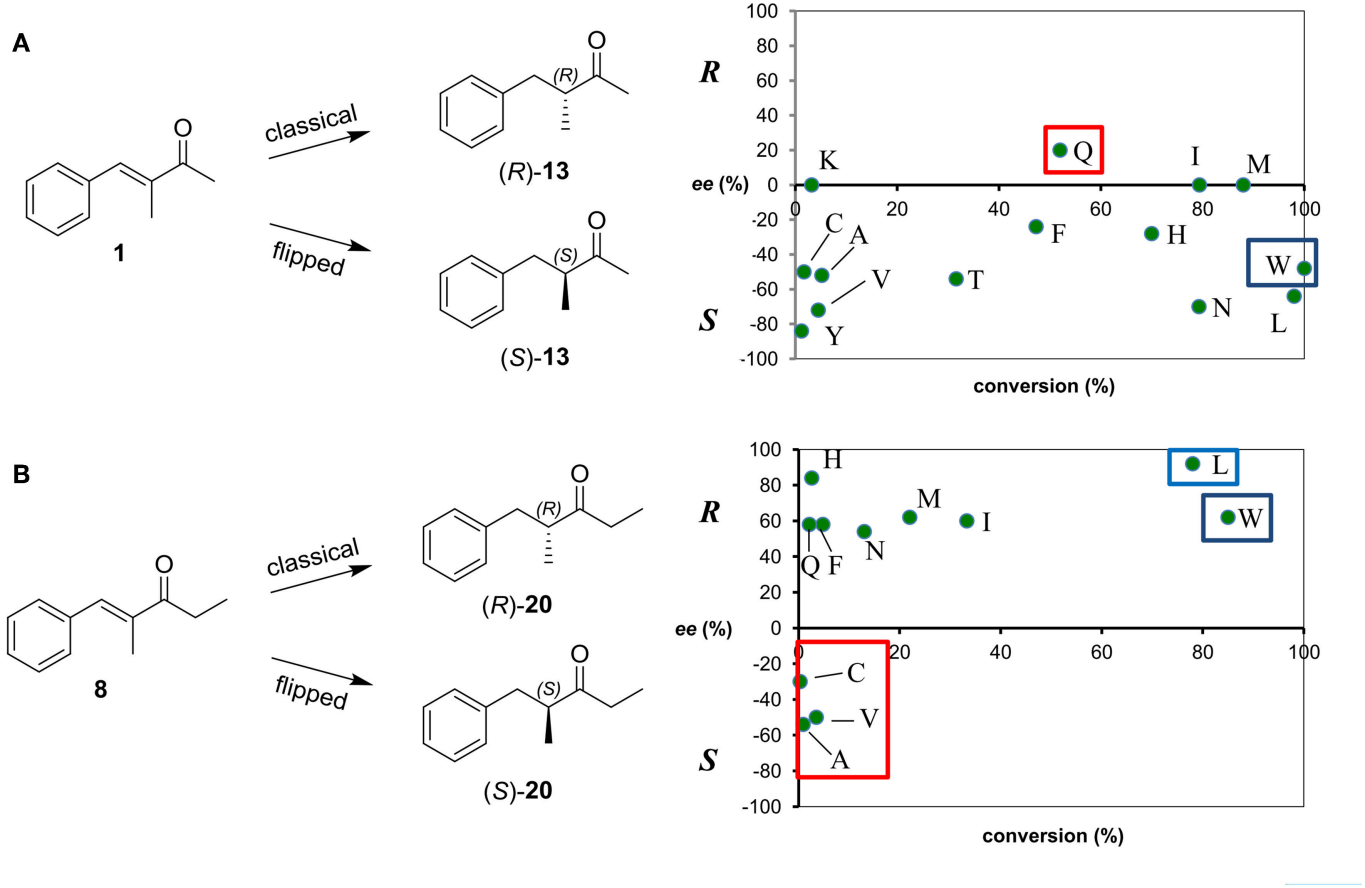
Conversion and stereoselectivity of Trp116 variants of OYE1 in the bioreduction of substrates **1 (A)** and **8 (B)**. Reactions were performed in triplicate, standard deviation of conversion and enantiomeric excess values was below 5%.

To try to improve these achievements, we considered also the screening of a library of OYE1 variants obtained by saturation mutagenesis at the W116 position. In fact, substitution at this “hot spot” position in OYE1 has often a dramatic impact in terms of stereoselectivity for the bioreduction of some substrates (Walton et al., 2011; Pompeu et al., 2013). In this case, given the higher number of variants, the screening was limited to representative methyl and ethyl ketones **1** and **8** (Figure 4, for the full set of data see Table S4).

As far as the enantioselectivity concerns, the effects of the W116 mutations were generally limited in the case of the bioreduction of the methyl derivative **1**, a loss of enantioselectivity being observed for the W116I, W116K, and W116M mutants, while a modest inversion of the stereopreference was shown only in the case of the W116Q variant [from 56% ee (*S*) with wild type OYE1 to 20% ee (*R*)] (Figure 4A).

On the contrary, a more pronounced enantioselectivity switch was observed when testing the W116A and W116V mutants and the ethyl substrate **8** (Figure 4B), the ee values of the reduced products going from 59% ee (*R*) with wild type OYE1 to 54 and 50% ee (*S*), respectively. Unfortunately, this effect on the selectivity was in both cases accompanied to a dramatic drop of the conversions. Among the other variants, it is worth of mentioning the W116L mutant which showed a significantly increased *R* selectivity (98% ee).

### Computational Studies and Screening of Double Mutants of OYE1

To gather information on the effect of the mutations at position 116 on the enantioselectivity, we performed a computational study of the docking mode of the ethyl ketone **8** on the wild type OYE1 and on the W116L, W116V, and W116A mutants. The structure of OYE1 was obtained according to a previously described procedure (Brenna et al., 2013a), whereas for the mutant enzymes we used the models obtained by the x-ray structure reported in the literature (PDB code 4K7V, 4GEW, and 4K8H) (Pompeu et al., 2013).

**Figure 5:**
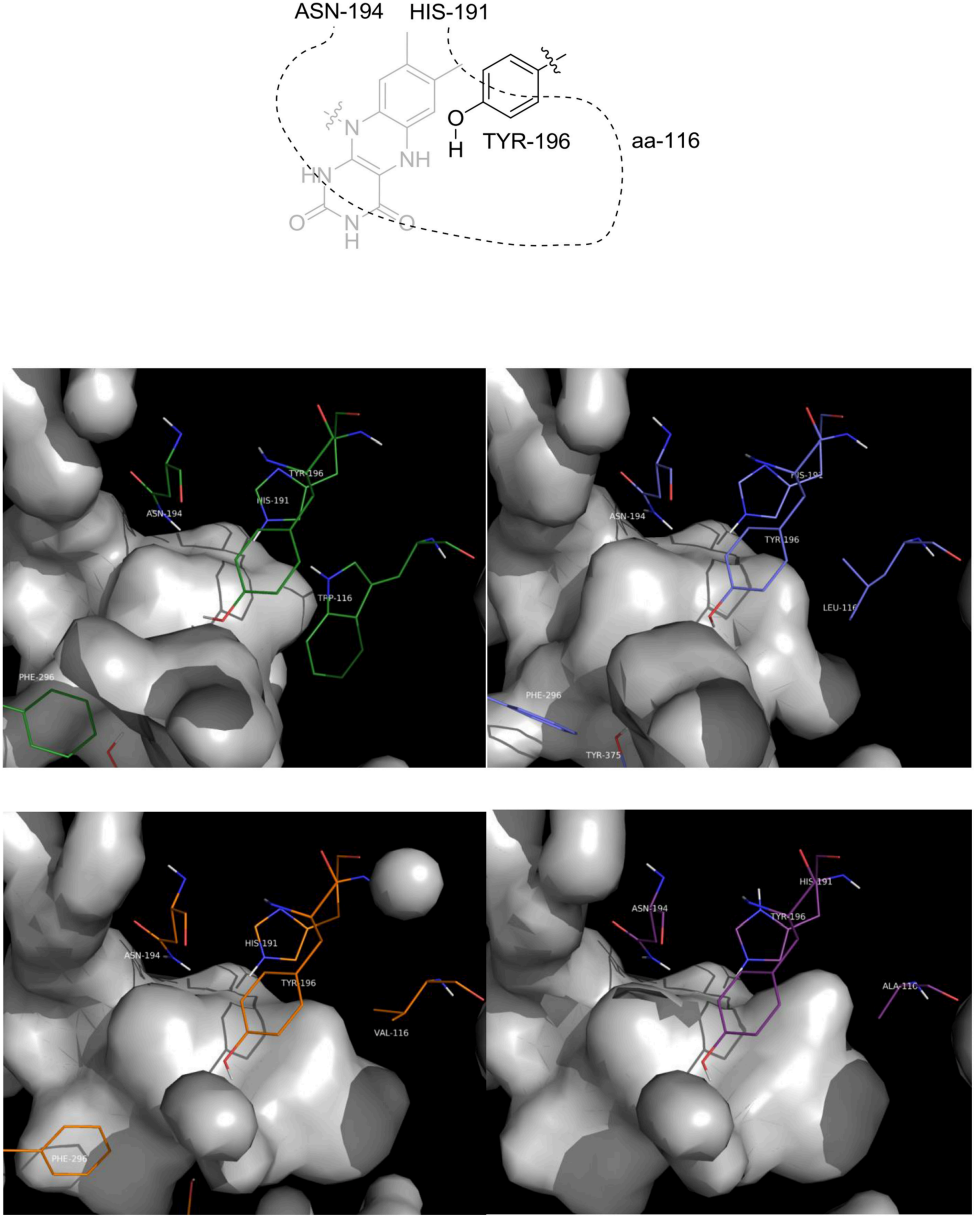
Picture of the inner cavity (gray surface) of the active site. Upper left: wild type OYE1; upper right: OYE1 W116L; lower left: OYE1 W116V; lower right: OYE1 W116A.

The first observation is that the shape and the volume of the active site, as expected, changed as a function of the residue at position 116 (aa-116, Figure 5): as the steric hindrance of the residue is reduced, from Trp to Ala, there is an increase of the available space in that part of the active site which is occupied by the ethyl moiety of the ketone when a flipped binding mode is adopted.

Docking were performed with Autodock, as implemented in the YASARA software. The structures of the substrate were prepared and optimized with Spartan'08. Both the *s-trans* and *s-cis* rotamers of the substrate have been considered in the docking study. For all the cases, the substrate is coordinated to the enzyme active site *via* hydrogen bonds between the carbonyl and the N194 and H191 residues. As a consequence, the substrate is activated toward the β-addition of hydride from the FMNH cofactor: finally a hydrogen atom is provided by the Y196 hydroxyl group, thus promoting the stereoselective reduction of the double bond.

**Figure 6:**
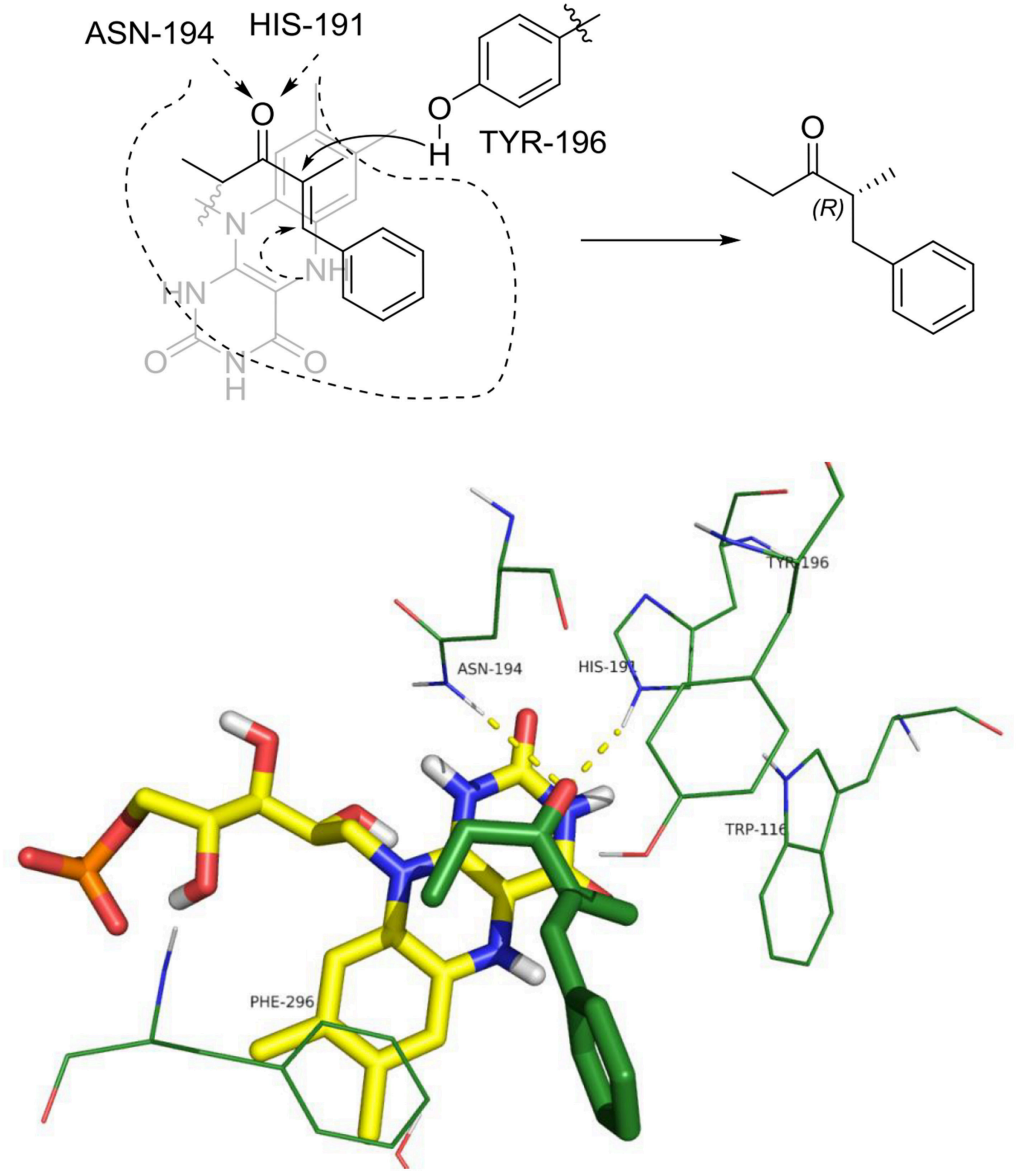
Binding mode of substrate **8** for wild type OYE1.

**Figure 7:**
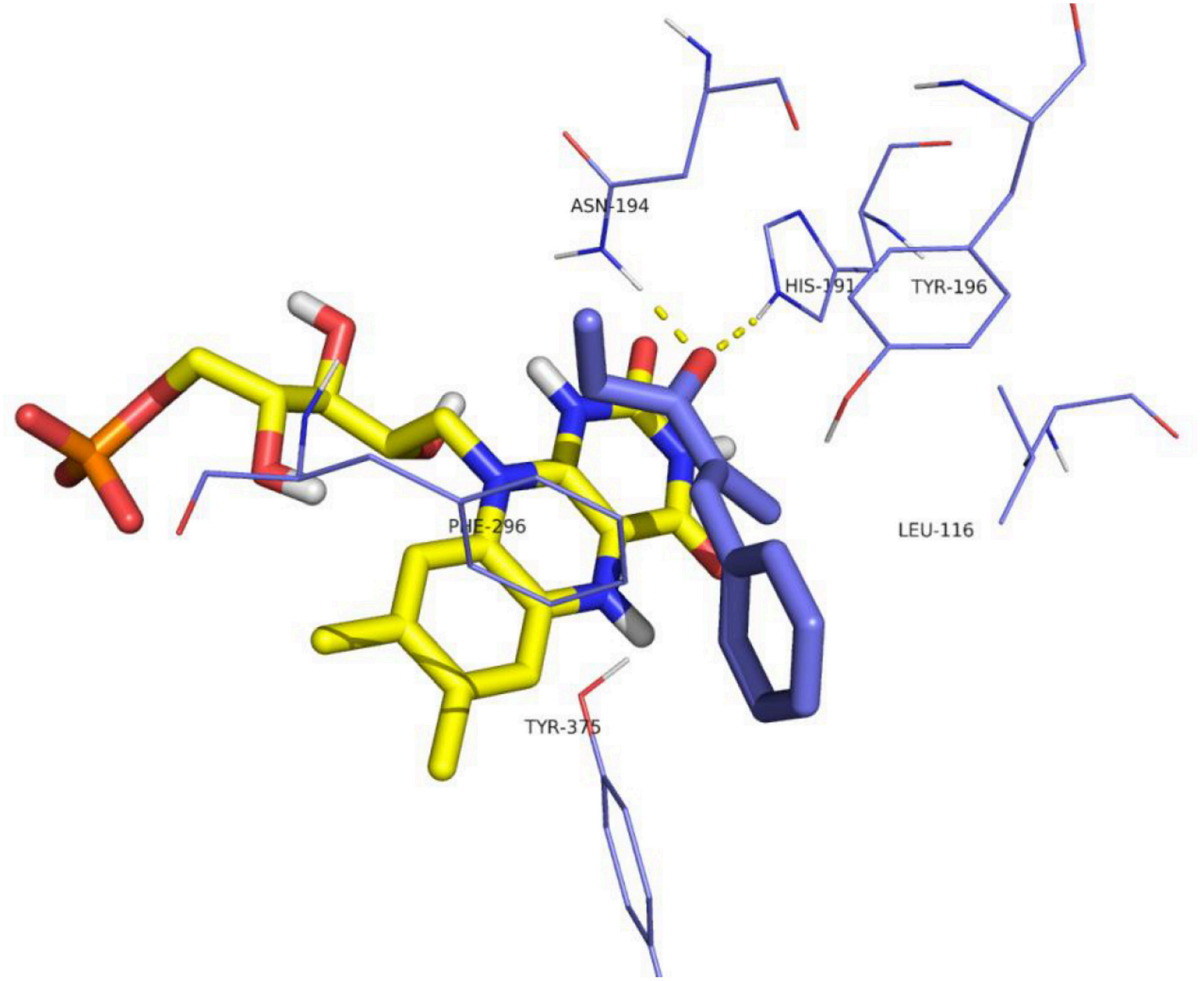
Binding mode of substrate **8** for OYE1 W116L.

The distances between the reactive C = C atoms of the substrate and the FMNH_2_ cofactor (4.4 Å) or the OH of Y196 (3.0 – 3.3Å) are very similar for all the cases, regardless the *s-trans* or *s-cis* conformation of the substrate. For each enzyme, the best pose has been considered according to the score which has been measured in kcal/mol as the binding energy (an higher score means a more stable complex). For OYE1 and for the W116L variant the calculated binding energy were 7.93 and 7.66 kcal/mol, respectively. Slightly higher values were obtained for W116V (8.91 kcal/mol) and for W116A (8.63 kcal/mol) mutants. In the case of the wild type OYE1 and the W116L mutant (Figures 6, 7, respectively), the most stable complex was obtained with the *s-trans* rotamer of the substrate in the classical binding mode. The *anti* hydrogen addition to the substrate in this binding mode resulted in the formation of the *(R)*-enantiomer of the corresponding saturated ketone, in accordance with the experimental results.

**Figure 8:**
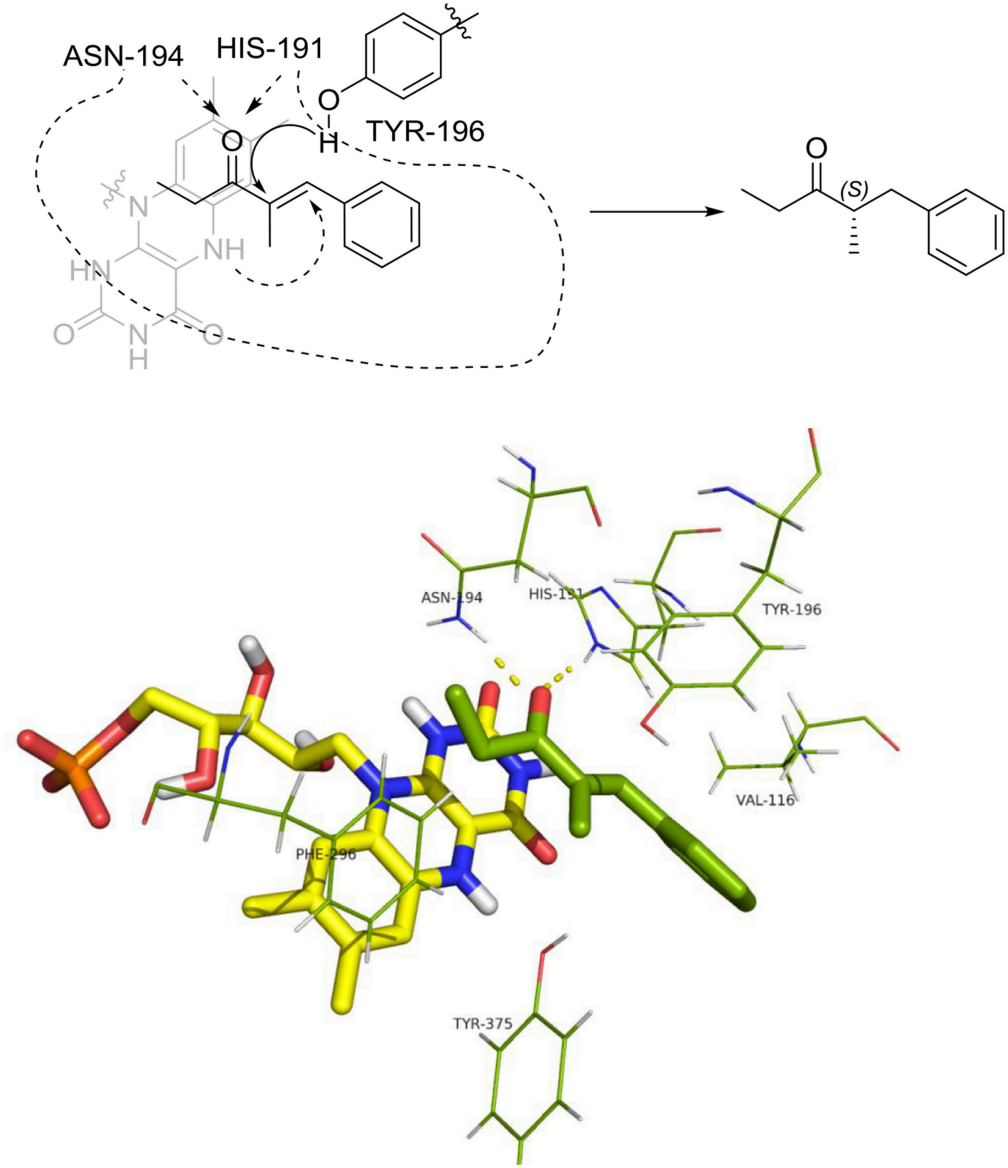
Binding mode of substrate **8** for OYE1 W116V.

**Figure 9:**
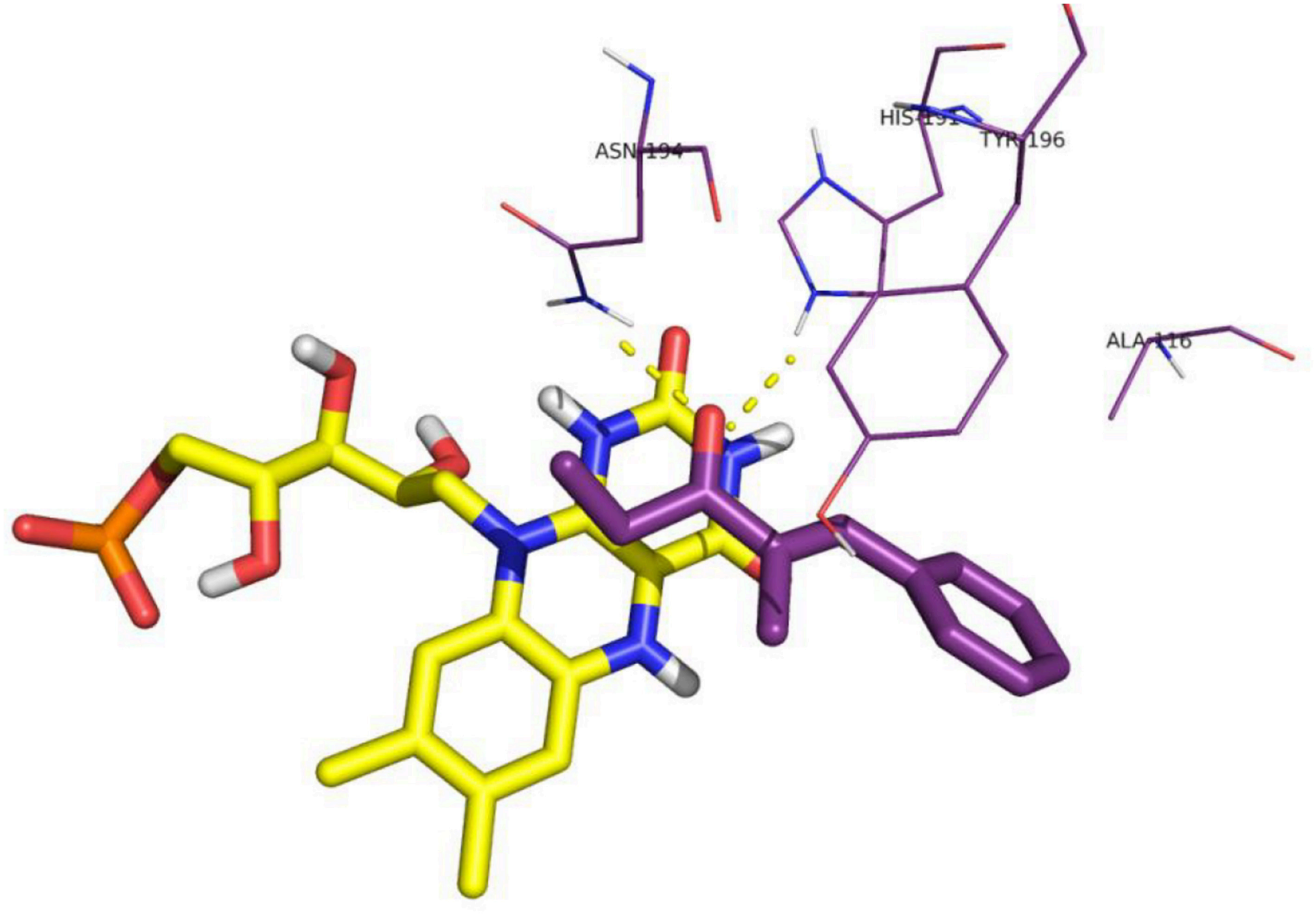
Binding mode of substrate **8** for OYE1 W116A.

When both W116V and W116A mutants were considered (Figures 8, 9, respectively), the lowest energy complex was obtained with a *s-cis* rotamer of the substrate in the flipped binding mode. In this case the Ala and Val residues are small enough to allow the placing of the bulky phenyl residue in the right portion of the active site. The result was a flipped binding mode, which produced the *(S)*-enantiomer of the saturated ketone upon *anti* hydrogen addition.

As mutations at both hot spots in position 296 and 116 of OYE1 had a similar effect in generating stereocomplementary variants, we wondered whether an additive effect might be observed in the double mutants or not. Therefore, two additional variants of OYE1, F296S/W116A and F296S/W116V, were obtained by site-directed mutagenesis and tested in the previously described bioreductions.

As shown in Table 1 (lines 5 and 6), also these two new variants showed a very good *S*-selectivity toward the methyl derivative **1** (90 and 94% ee, respectively), while the conversions resulted decreased in the respect of the wild type enzyme, but less dramatically than with the single W116A and W116V mutants (Figure 4A).

With our delight, a very positive effect of the introduction of the double mutations was observed with the ethyl ketones **8**, **10**, and **11**. In fact, not only the enantioselectivity switch observed with the single mutants was retained, but, in both cases, the selectivity significantly improved, even exceeding that observed with OYE3, e.g., with substrate **10**, 98% ee with any of the double mutants and 95% ee with OYE3. In the case of **8** and **10**, the conversions improved as well, both in respect of the single F296S and W116A/V mutants.

### Effect of the Mutations in the Bioreduction of Carvone Enantiomers

Finally, the effect of the F296S and F296S/W116A/V mutations on the activity and stereoselectivity in the bioreduction of the typical ER substrates (*S*)- and (*R*)-carvone (Padhi et al., 2009; Nett et al., 2017; Powell et al., 2018) was investigated (Table 4).

**Table 4:** Bioreduction of (*S*)- and (*R*)-carvone by wild type and mutant ERs.

**Entry**	**ER**	**Substrate**			
		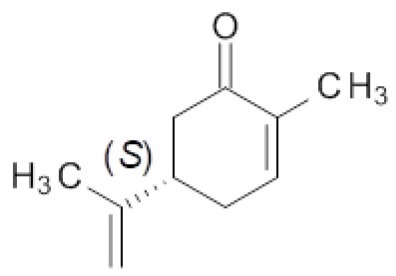		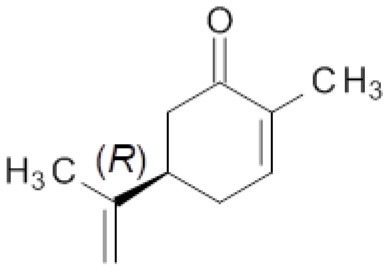	
		**25**		**26**	
		***c* [%]^**a**^**	***de* [%]^**a**^**	***c* [%]^**a**^**	***de* [%]^**a**^**
1	OYE1	59	83 (2*R*,5*S*)	>99	>99 (2*R*,5*R*)
2	OYE2	50	80 (2*R*,5*S*)	>99	>99 (2*R*,5*R*)
3	OYE3	25	84 (2*R*,5*S*)	>99	>99 (2*R*,5*R*)
4	OYE1 F296S	34	82 (2*R*,5*S*)	>99	>99 (2*R*,5*R*)
5	OYE1 F296S/W116A	27	96 (2*S*,5*S*)	3	28 (2*S*,5*R*)
6	OYE1 F296S/W116V	17	96 (2*S*,5*S*)	4	18 (2*R*,5*R*)

a *Conversion and diastereoisomeric excess values determined by GC-MS. Reactions were performed in triplicate, standard deviation of conversion and diastereomeric excess values was below 5%*.

For both substrate enantiomers, the F296S substitution (line 4) had no significant effect in the outcome of the reactions in the respect of the results obtained with the wild type OYE1 (line 1). Indeed, these results are not surprising since similar performances could be obtained with OYE3 as well (line 3).

Instead, as far as (*S*)-carvone concerns (substrate **25**), both double mutants showed a clear diastereoselectivity switch, from 83% de (2*R*,5*S*) with wild type OYE1 to 96% de (2*S*,5*S*) with the variants (lines 5 and 6). This result is consistent with those previously obtained with the single W116A/V OYE1 mutants (Pompeu et al., 2013) and could be similarly explained by the occurrence for this substrate of a flipped binding orientation in the variants instead of the classical binding mode in OYE1. When tested on the (*R*) enantiomer (substrate **26**), both double mutants showed a dramatic drop of the conversions, from quantitative to 3-4% (lines 5 and 6). As far as the stereoselectivity concerns, the behavior of the F296S/W116A/V OYE1 mutants resembled more that of the W116A/V OYE3 variants than of the corresponding OYE1 single mutants (Powell et al., 2018). In fact, OYE1 F296S/W116A displayed a change of selectivity by preferring the formation of the 2*S*,5*R* diastereomer (line 5), while OYE1 F296S/W116V retained the same stereopreference shown by wild type OYE1 (line 6). In both cases, however, the diastereoselectivities were significantly lower than those observed with both the wild type OYE1-3 enzymes and OYE1 F296S.

## Discussion

The screening of the library of wild type ERs in the bioreduction of α,β-unsaturated-β-arylketones **1**-**8**, **10**, and **11** (Table 2) shows that the presence of an ethyl instead of a methyl group linked to the carbonyl moiety (substrates **8**, **10** and **11** vs. **1**–**7**) causes a switch from (*S*) to (*R*) enantioselectivity in most reactions. The only exceptions are the transformations catalyzed by OYE3 and LtB4DH, affording invariably the (*S*)-enantiomer of the reduced product. These results clearly highlight the odd behavior of OYE3 in controlling the stereochemistry of the hydrogenation of this class of substrates with respect to the most common wild type OYEs. According to our data, the (*S*)-selectivity in the reduction of both methyl and ethyl ketones is shared by OYE3 with the non-OYE reductase from *Rattus norvegicus*.

The understanding of the stereochemical outcome of OYE3 – mediated reductions of ethyl ketones **8** -**11** is still more puzzling if compared to that of the transformations catalyzed by OYE 1 (Table 2), because of the high structural similarity between the two enzymes. The only difference in their active site is the presence in position 296 of a Ser residue in OYE3 and a Phe residue in OYE1. Stewart et al. tentatively attributed the different enantioselectivity shown by OYE 1 and OYE3 in the reduction of certain substrates to the different conformational preferences of loop 6 of the two proteins in solution. Molecular dynamics simulations highlighted that the conformation of loop 6 in OYE 1 changes from crystal to solution. In solution, loop 6 of OYE 1 closes down over the active site, making it interact closely with bound substrates. On the contrary, loop 6 of OYE 3 maintains in solution the same conformation observed in the crystal. The loop does not close over the active site of OYE 3, there are fewer protein—substrate contacts and substrate binding is less influenced by the residues of the active site (Powell et al., 2018).

When we removed the only structural difference between the active sites of OYE1 and OYE3 by preparing OYE1 F296S variant, a switch from (*R*)- to (*S*)-reduced products was obtained starting from ethyl ketones **8**, **10**, and **11** (Table 3, entry 4). Interestingly, the same switch from (*R*) to (*S*)- selectivity was observed when the reduction of ketone **8** was catalyzed by OYE1 mutants showing residues of low steric hindrance in position 116, such Ala, Cys, or Val, in place of the Trp unit of the wild type (Table S4 and Figure 4B). The (*R*)-enantiomer was instead obtained through a classical binding mode with those variants characterized by a residue in position 116 with large Van der Waals volume (>125 Å^3^), including the wild type (for tryptophan V = 163 Å^3^) and with the exception of W116N (for asparagine V = 96 Å^3^) (Darby and Creighton, 1993).

The critical role played by the W116 residue in controlling the stereochemistry of OYE1-catalyzed hydrogenations of substrates showing certain substitution patterns has been already discussed in the literature (Pompeu et al., 2013; Brenna et al., 2015a), and employed to orient the stereochemical outcome of the reaction. As for the class of unsaturated compounds herein investigated, the reduction of ethyl ketone **8** is more influenced by the nature of the amino acid in position 116 than that of the methyl ketone **1**. The data collected in Table S4 and depicted in Figure 4A show that the hydrogenation of methyl ketone **1** affords the (*S*)*-*enantiomer of the reduced product with most OYE1 W116X enzymes. The (*R*)-enantiomer is obtained only using the W116Q variant, but with poor enantioselectivity. Thus, the ethyl group linked to the carbonyl makes the unsaturated ketone a more effective probe of the nature of the amino acid 116.

When the two key mutations of OYE1 protein chain favoring (*S*)-selectivity (F296S and W116A/V) were combined, the preference for the (*S*)-enantiomer of the reduced product indeed increased in the transformation of the three ethyl ketones **8**, **10**, and **11** (Table 3, entries 5 and 6 to be compared with entry 4 of the same Table and entries 1 and 18 of Table S4).

The increase of available free space around position 116 by the insertion of an amino acid residue which is less hindered than Trp, and the effect on loop 6 conformation possibly produced by F296S mutation definitely favor a flipped binding mode for the substrate and promote the obtainment of the (*S*)-enantiomer of the corresponding saturated ketone in high optical purity.

As for carvone enantiomers, their reduction occurs in a classical binding mode both with OYE1 and OYE3. Thus, their accommodation in the active site of the two enzymes is not influenced by the nature of the amino acid in position 296. When the two double mutants of OYE1 are employed, the results obtained with (*S*)-carvone are in agreement with those observed for OYE1 W116A/V, as if the F296S mutation was uninfluential. By contrast, in the case of (*R*)-carvone the double mutants of OYE1 behave as the single mutants in position 116 of OYE3.

## Author Contributions

MC and FP performed the bioreduction screening. EF designed and produced the mutants. AS carried out the computational studies. FG, SR, AS, EB, and DM analyzed the data and wrote the paper.

### Conflict of Interest Statement

The authors declare that the research was conducted in the absence of any commercial or financial relationships that could be construed as a potential conflict of interest.
