# Supplementary material for: Stereoselectivity Switch in the Reduction of α-Alkyl-β-Arylenones by Structure-Guided Designed Variants of the Ene Reductase OYE1
Source: Front Bioeng Biotechnol. 2019 Apr 26;7:89. doi: 10.3389/fbioe.2019.00089 (PMC6497740; doi:10.3389/fbioe.2019.00089)
Supplement: Supplementary file 1 [file Data_Sheet_1.PDF]

Supplementary Material

Stereoselectivity Switch in the Reduction of α-Alkyl-β-arylenones by Structure-guided Designed Variants of the Ene Reductase OYE1

Michele Crotti[1], Fabio Parmeggiani[1], Erica Elisa Ferrandi[2], Francesco G. Gatti[1], Alessandro Sacchetti[1], Sergio Riva[2], Elisabetta Brenna[1*], Daniela Monti[2*]

*** Correspondence:** Elisabetta Brenna: mariaelisabetta.brenna@polimi.it; Daniela Monti: daniela.monti@icrm.cnr.it

Table S1. Primers used for site directed mutagenesis

**Primer name**	**Primer sequence (5'to3')**
F296S OYE1	5'-cctcgtgtaactaacccaagcttgactgaaggggaggg-3'
F296S OYE1 antisense	5'-ccctccccttcagtcaagcttgggttagttacacgagg-3'
W116A-F296S OYE1	5'-agaaatcgttcgtttgggttcagttagcggttttgggttggg-3'
W116A-F296S OYE1 antisense	5'-cccaacccaaaaccgctaactgaacccaaacgaacgatttct-3'
W116V-F296S OYE1	5'-agaaatcgttcgtttgggttcagttagtggttttgggttggg-3'
W116V-F296S OYE1 antisense	5'-cccaacccaaaaccactaactgaacccaaacgaacgatttct-3'

Table S2. Sources and sequences of ene reductases used in this study.

ER	Source	Species	UniProtKB accession number
OYE1	Yeast	*Saccharomyces pastorianus*	Q02899
OYE2	Yeast	*Saccharomyces cerevisiae*	Q03558
OYE3	Yeast	*Saccharomyces cerevisiae*	P41816
NemA	Bacterium	*Escherichia coli*	P77258
OPR1	Plant	*Arabidopsis thaliana*	Q8LAH7
OYE2.6	Yeast	*Scheffersomyces stipitis*	A3LT82
PpNemA	Bacterium	*Pseudomonas putida*	Q88I29
LeOPR1	Plant	*Solanum lycopersicum*	Q9XG54
KmOYE	Yeast	*Kluyveromyces marxianus*	Q6I7B7
LtB4DH	Animal	*Rattus norvegicus*	P97584
PpOYE	Bacterium	*Pseudomonas putida*	Q88K07

Table S3. Identity percentages of ERs from the OYE family at the amino acid level. Data were obtained by sequence alignment by using the BLASTP tool. OYEs from yeasts are highlighted in purple, bacterial enzymes in light blue, while OYEs from plants in salmon.

% identity	**OYE1**	**OYE 2**	**OYE 3**	**OYE2.6**	**KmOYE**	**Nema**	**PpOYE**	**PpNema**	**OPR1**	**LeOPR**
**OYE1**	100	92	80	44	69	37	33	34	35	37
**OYE2**	92	100	82	42	72	37	36	36	39	39
**OYE 3**	80	82	100	42	71	39	34	37	37	39
**OYE 2.6**	44	42	42	100	43	36	35	35	35	36
**KmOYE**	69	72	68	43	100	38	34	36	34	37
**Nema**	37	37	39	36	38	100	46	69	41	42
**PpOYE**	33	36	34	35	34	46	100	44	38	39
**PpNema**	34	36	37	35	36	69	44	100	39	42
**OPR 1**	35	39	37	35	34	41	38	39	100	68
**LeOPR**	37	39	38	36	35	42	42	42	68	100

Table S4. Screening of W116 variants of OYE1 in the reduction of α,β-unsaturated methyl and ethyl ketones **1** and **8**.

Enzyme	Substrate			
	**1**		**8**	
	c [%][a)]	*ee* [%][b)]	*c* [%][a)]	*ee* [%][b)]
OYE1 wild-type	>99	56 (*S*)	67	59 (*R*)
OYE1 W116A	5.2	52 (*S*)	0.9	54 (*S*)
OYE1 W116C	1.7	50 (*S*)	0.3	30 (*S*)
OYE1 W116D	-	n.d[c)]	-	n.d[c)]
OYE1 W116E	0.3	n.d[c)]	-	n.d[c)]
OYE1 W116F	47.3	24 (*S*)	4.8	58 (*R*)
OYE1 W116G	0.2	n.d[c)]	-	n.d[c)]
OYE1 W116H	70	28 (*S*)	2.6	84 (*R*)
OYE1 W116I	79.4	*rac*	33.3	60 (*R*)
OYE1 W116K	3.2	*rac*	-	n.d[c)]
OYE1 W116L	98	64 (*S*)	78	92 (*R*)
OYE1 W116M	88	*rac*	22	62 (*R*)
OYE1 W116N	79	70 (*S*)	13	54 (*R*)
OYE1 W116P	-	n.d[c)]	-	n.d[c)]
OYE1 W116Q	52	20 (*R*)	2.13	58 (*R*)
OYE1 W116R	-	n.d[c)]		n.d[c)]
OYE1 W116S	-	n.d[c)]	-	n.d[c)]
OYE1 W116T	31.6	54 (*S*)	-	n.d[c)]
OYE1 W116V	4.5	72 (*S*)	3	50 (*S*)
OYE1 W116Y	1.2	84 (*S*)	3.5	62 (*R*)

[a)] Conversion values determined by GC-MS. [b)] Enantiomeric excess values determined by GC on a chiral stationary phase. **[c)]** n.d.: Not determined, below detection limit.

Table S5. Chiral GC methods and retention times (t_R) of bioreduction products.

Product	Method[a)]	t_R (*R*)	t_R (*S*)
**13**	1	19.6 min	20.4 min
**14**	2	43.0 min	43.7 min
**15**	3	38.4 min	39.0 min
**16**	1	43.4 min	44.1 min
**17**	1	20.3 min	20.8 min
**18**	5	29.8 min	30.1 min
**19**	6	27.9 min	28.1 min
**20**	3	21.9 min	22.8 min
**21**	3	35.7 min	36.5 min
**22**	3	43.1 min	43.8 min
**23**	4	37.3 min	37.6 min

[a)] Chiral GC methods (analytical conditions as described in the Materials and Methods if not stated otherwise): Method 1 (for compounds **13**, **16**, **17**): 75 °C (1 min) / 1 °C min^{-1} / 125 °C (0 min) / 30 °C min^{-1} / 180°C (2 min). Method 2 (for compound **14**): 60 °C (1 min) / 1 °C min^{-1} / 108 °C (0 min) / 30 °C min^{-1} / 180°C (2 min). Method 3 (for compounds **15, 20, 21, 22**): 75 °C (1 min) / 1 °C min^{-1} / 135 °C (0 min) / 30 °C min^{-1} / 180°C (2 min). Method 4 (for compound **23**): 60 °C (1 min) / 2 °C min^{-1} / 150 °C (0 min) / 30 °C min^{-1} / 180°C (2 min). Method 5 (for compound **18**): 90 °C (1 min) / 2 °C min^{-1} / 160 °C (0 min) / 30 °C min^{-1} / 180°C (2 min). Method 6 (for compound **19**, on a Mega DAcTBSil.BetaCDEX (25 m × 0.25 mm × 0.25 μm) column): 70 °C (0 min) / 2 °C min^{-1} / 136 °C (0 min) / 30 °C min^{-1} / 220°C (10 min).

Synthesis of α,β-unsaturated enones 1-12 and of racemic saturated compound 13-23

The synthesis of α,β-unsaturated enones **1-12** and of racemic saturated compounds **13-23** was carried out according to the procedures already described in reference 1.

Characterization data of saturated ketones 13-23

3-Methyl-4-phenyl-2-butanone (13): Colorless liquid, 98% purity by GC (t_R 13.30 min); chiral GC (method 1): t_R (*R*) 19.6 min, t_R (*S*) 20.4 min; ^{1}H NMR (400 MHz, $CDCl_3$): [2] δ = 7.23-7.30 (m, 2H, arom.), 7.11-7.21 (m, 3H, arom.), 2.99 (dd, *J* = 6.6, 13.2 Hz, 1H, ArC*H*H), 2.77-2.87 (m, 1H, C*H*), 2.56 (dd, *J* = 7.7, 13.6 Hz, 1H, ArCH*H*), 2.07 (s, 3H, COCH_3), 1.08 (d, *J* = 7.0 Hz, 3H, CH_3) ppm; ^{13}C NMR (100.6 MHz, $CDCl_3$): δ = 211.8, 139.7, 128.9, 128.4, 126.2, 48.7, 38.9, 28.7, 16.1 ppm; MS: m/z (%) = 162 $[M]^+$ (41), 147 (33), 119 (26), 91 (100), 43 (29).

4-(2-Methoxyphenyl)-3-methyl-2-butanone (14): Colorless liquid, 99% purity by GC (t_R 17.77 min); chiral GC (method 2): t_R (*R*) 43.0 min, t_R (*S*) 43.7 min; ^{1}H NMR (400 MHz, $CDCl_3$):[2] δ = 7.14-7.21 (m, 1H, arom.), 7.04-7.08 (m, 1H, arom.), 6.81-6.88 (m, 2H, arom.), 3.80 (s, 3H, OCH_3), 2.99 (dd, *J* = 6.5, 13.3 Hz, 1H, ArC*H*H), 2.84-2.93 (m, 1H, C*H*), 2.56 (dd, *J* = 7.5, 13.3 Hz, 1H, ArCH*H*), 2.09 (s, 3H, COCH_3), 1.04 (d, *J* = 7.2 Hz, 3H, CH_3) ppm; ^{13}C NMR (100.6 MHz, $CDCl_3$): δ = 212.2, 157.4, 130.8, 127.9, 127.5, 120.3, 110.2, 55.1, 46.8, 33.8, 28.4, 15.9 ppm; MS: m/z (%) = 192 $[M]^+$ (44), 177 (6), 149 (11), 121 (100), 108 (19), 91 (56).

4-(3-Methoxyphenyl)-3-methyl-2-butanone (15): Colorless liquid, 93% purity by GC (t_R 18.50 min); chiral GC (method 3): t_R (*R*) 38.4 min, t_R (*S*) 39.0 min; ^{1}H NMR (400 MHz, $CDCl_3$):[2] δ = 7.15-7.21 (m, 1H, arom.), 6.68-6.76 (m, 3H, arom.), 3.77 (s, 3H, OCH_3), 2.97 (dd, *J* = 6.5, 13.4 Hz, 1H, ArC*H*H), 2.75-2.89 (m, 1H, C*H*), 2.53 (dd, *J* = 7.9, 13.7 Hz, 1H, ArCH*H*), 2.08 (s, 3H, COCH_3), 1.09 (d, *J* = 6.9 Hz, 3H, CH_3) ppm; ^{13}C NMR (100.6 MHz, $CDCl_3$): δ = 214.3, 159.6, 141.4, 129.2, 121.2, 114.6, 111.5, 55.0, 47.6, 39.2, 34.9, 16.5 ppm; MS: m/z (%) = 192 $[M]^+$ (68), 177 (8), 149 (100), 121 (92), 91 (26).

4-(4-Methoxyphenyl)-3-methyl-2-butanone (16): Colorless liquid, 98% purity by GC (t_R 18.84 min); chiral GC (method 1): t_R (*R*) 43.4 min, t_R (*S*) 44.1 min; ^{1}H NMR (400 MHz, $CDCl_3$):[2] δ = 7.02-7.10 (m, 2H, arom.), 6.79-6.84 (m, 2H, arom.), 3.77 (s, 3H, OCH_3), 2.92 (dd, *J* = 7.0, 13.6 Hz, 1H, ArC*H*H), 2.73-2.84 (m, 1H, C*H*), 2.51 (dd, *J* = 7.7, 13.6 Hz, 1H, ArCH*H*), 2.07 (s, 3H, COCH_3), 1.07 (d, *J* = 7.0 Hz, 3H, CH_3) ppm; ^{13}C NMR (100.6 MHz, $CDCl_3$): δ = 212.0, 158.1, 131.7, 129.8, 113.8, 55.1, 48.9, 38.1, 28.7, 16.1 ppm; MS: m/z (%) = 192 $[M]^+$ (24), 177 (110), 121 (100), 91 (9).

3-Benzyl-2-pentanone (17): Colorless liquid, 94% purity by GC (t_R 15.31 min); chiral GC (method 1): t_R (*R*) 20.3 min, t_R (*S*) 20.8 min; ^{1}H NMR (400 MHz, $CDCl_3$):[2] δ = 7.23-7.30 (m, 2H, arom.), 7.11-7.21 (m, 3H, arom.), 2.88 (dd, *J* = 7.2, 12.9 Hz, 1H, ArC*H*H), 2.64-2.88 (m, 2H, C*H* + ArCH*H*), 1.99 (s, 3H, COCH_3), 1.46-1.72 (m, 2H, CH_2CH$_3$), 0.89 (t, *J* = 7.6 Hz, 3H, CH$_2$CH_3) ppm; ^{13}C NMR (100.6 MHz, $CDCl_3$): δ = 212.1, 139.7, 128.8, 128.4, 126.2, 56.1, 37.4, 30.0, 24.5, 11.5 ppm; MS: m/z (%) = 176 $[M]^+$ (8), 161 (5), 147 (82), 129 (10), 117 (16), 91 (100).

3-(3-Nitrobenzyl)-2-pentanone (18): Colorless liquid, 97% purity by GC (t_R 22.31 min); chiral GC (method 5): t_R (*R*) 29.8 min, t_R (*S*) 30.1 min; ^{1}H NMR (400 MHz, $CDCl_3$):[3] δ = 7.98-8.09 (m, 2H, arom.), 7.38-7.52 (m, 2H, arom.), 2.98-3.08 (m, 1H, ArC*H*H), 2.70-2.87 (m, 2H, C*H* + ArCH*H*), 2.06

(s, 3H, COCH_3), 1.62-1.77 (m, 1H, C*H*HCH$_3$), 1.48-1.63 (m, 1H, CH*H*CH$_3$), 0.93 (t, *J* = 7.3 Hz, 2H, CH$_2$CH_3) ppm; ^{13}C NMR (100.6 MHz, CDCl$_3$): δ = 210.9, 148.3, 142.0, 135.2, 129.3, 123.6, 121.4, 55.5, 36.2, 30.0, 24.5, 11.3 ppm; MS: m/z (%) = 192 [M–29]$^+$ (91), 161 (100), 136 (62), 117 (45), 90 (53).

3-Benzyl-2-heptanone (19): Colorless liquid, 96% purity by GC (t_R 19.35 min); chiral GC (method 6): t_R (*R*) 27.9 min, t_R (*S*) 28.1 min; ^{1}H NMR(400 MHz, CDCl$_3$):[4] δ = 7.23-7.29 (m, 2H, arom.), 7.10-7.21 (m, 3H, arom.), 2.75-2.93 (m, 2H, ArC*H*H + C*H*), 2.67 (dd, *J* = 5.7, 12.9 Hz, 1H, ArC*H*H), 1.98 (s, 3H, COCH_3), 1.58-1.67 (m, 1H, C*H*H), 1.41-1.50 (m, 1H, CH*H*), 1.19-1.33 (m, 4H, CH_2CH_2), 0.87 (t, *J* = 6.8 Hz, 3H, CH$_2$CH_3) ppm; ^{13}C NMR (100.6 MHz, CDCl$_3$): δ = 212.2, 139.7, 128.8, 128.4, 126.2, 54.7, 37.9, 31.4, 30.0, 29.4, 22.7, 13.8 ppm; MS: m/z (%) = 204 [M]$^+$ (6), 147 (100), 129 (11), 117 (16), 105 (19), 91 (96).

2-Methyl-1-phenyl-3-pentanone (20): Colorless liquid, 93% purity by GC (t_R 14.97 min); chiral GC (method 3): t_R (*R*) 21.9 min, t_R (*S*) 22.8 min; ^{1}H NMR (400 MHz, CDCl$_3$):[2] δ = 7.15-7.32 (m, 5H, arom.), 3.00 (dd, *J* = 7.1, 13.1 Hz, 1H, ArC*H*H), 2.83-2.90 (m, 1H, C*H*), 2.58 (dd, *J* = 7.2, 13.3 Hz, 1H, ArCH*H*), 2.21-2.52 (m, 2H, CH_2CH$_3$), 1.10 (d, *J* = 6.9 Hz, 3H, CH_3), 1.00 (t, *J* = 7.3 Hz, 3H, CH$_2$CH_3) ppm; ^{13}C NMR (100.6 MHz, CDCl$_3$): δ = 214.8, 139.9, 128.9, 128.4, 128.1, 126.2, 47.9, 39.3, 35.2, 16.6, 7.6 ppm; MS: m/z (%) = 176 [M]$^+$ (29), 147 (47), 119 (39), 91 (100).

1-(2-Methoxyphenyl)-2-methyl-3-pentanone (21): Colorless liquid, 96% purity by GC (t_R 19.17 min); chiral GC (method 3): t_R (*R*) 35.7 min, t_R (*S*) 36.5 min; ^{1}H NMR (400 MHz, CDCl$_3$): δ = 7.14-7.20 (m, 1H, arom.), 7.02-7.07 (m, 1H, arom.), 6.80-6.86 (m, 2H, arom.), 3.81 (s, 3H, OCH_3), 2.85-2.99 (m, 2H, ArC*H*H + C*H*), 2.51-2.63 (m, 1H, ArCH*H*), 2.25-2.47 (m, 2H, CH_2CH$_3$), 1.03 (d, *J* = 6.8 Hz, 3H, CH_3), 0.98 (t, *J* = 7.2 Hz, 3H, CH$_2$CH_3) ppm; ^{13}C NMR (100.6 MHz, CDCl$_3$): δ = 215.0, 157.5, 130.8, 128.1, 127.5, 120.2, 110.2, 55.1, 45.6, 34.8, 34.1, 16.2, 7.6 ppm; MS: m/z (%) = 206 [M]$^+$ (53), 177 (18), 149 (22), 121 (100), 91 (40).

1-(3-Methoxyphenyl)-2-methyl-3-pentanone (22): Colorless liquid, 93% purity by GC (t_R 19.74 min); chiral GC (method 3): t_R (*R*) 43.1 min, t_R (*S*) 43.8 min; ^{1}H NMR (400 MHz, CDCl$_3$): δ = 7.13-7.19 (m, 1H, arom.), 6.65-6.75 (m, 3H, arom.), 3.76 (s, 3H, OCH_3), 2.94 (dd, *J* = 7.2, 13.4 Hz, 1H, ArC*H*H), 2.77-2.88 (m, 1H, C*H*), 2.53 (dd, *J* = 7.2, 13.4 Hz, 1H, ArCH*H*), 2.42 (dq, *J* = 7.2, 17.5 Hz, 1H, C*H*HCH$_3$), 2.26 (dq, *J* = 7.2,17.8 Hz, 1H, CH*H*CH$_3$), 1.07 (d, *J* = 6.9 Hz, 3H, CH_3) 0.97 (t, *J* = 7.2 Hz, 3H, CH$_2$CH_3) ppm; ^{13}C NMR (100.6 MHz, CDCl$_3$): δ = 214.3, 159.6, 141.4, 129.2, 121.2, 114.6, 111.5, 55.0, 47.6, 39.2, 34.9, 16.5, 7.5 ppm; MS: m/z (%) = 206 [M]$^+$ (55), 149 (100), 121 (76), 91 (19).

1-(4-Methoxyphenyl)-2-methyl-3-pentanone (23): Colorless liquid, 96% purity by GC (t_R 20.01 min); chiral GC (method 4): t_R (*R*) 37.3 min, t_R (*S*) 37.6 min; ^{1}H NMR (400 MHz, CDCl$_3$): δ = 7.02-7.07 (m, 2H, arom.), 6.78-6.84 (m, 2H, arom.), 3.77 (s, 3H, OCH_3), 2.90 (dd, *J* = 7.5, 13.7 Hz, 1H, ArC*H*H), 2.74-2.85 (m, 1H, C*H*), 2.51 (dd, *J* = 7.2, 13.3 Hz, 1H, ArCH*H*), 2.41 (dq, *J* = 7.5, 7.8 Hz, 1H, C*H*HCH$_3$), 2.25 (dq, *J* = 7.2, 17.8 Hz, 1H, CH*H*CH$_3$), 1.06 (d, *J* = 6.8 Hz, 3H, CH_3), 0.97 (t, *J* = 7.2 Hz, 3H, CH$_2$CH_3) ppm; ^{13}C NMR (100.6 MHz, CDCl$_3$): δ = 214.9, 158.0, 131.8, 129.8, 113.8, 55.2, 48.1, 38.4, 35.2, 16.5, 7.6 ppm; MS: m/z (%) = 206 [M]$^+$ (18), 177 (9), 149 (8), 121 (100), 91 (11).

References

1. E. Brenna, S. L. Cosi, E. E Ferrandi, F. G. Gatti, D. Monti, F. Parmeggiani, A. Sacchetti, *Org. Biomol. Chem*. 2013, *11*, 2988-2996.

2. S.-M. Lu, C. Bolm, *Angew. Chem. Int. Ed.* **2008**, *47*, 8920-8923.

3. Y. Kawai, K. Saitou, K. Hida, D. H. Dao, A. Ohno, *Bull. Chem. Soc. Jpn.* **1996**, *69*, 2633-2638.

4. A. G. Myers, B. H. Yang, H. Chen, L. McKinstry, D. J. Kopecky, J. L. Gleason, *J. Am. Chem. Soc.* **1997**, *119*, 6496-6511.
